# Geographical and climatic limits of needle types of one- and two-needled pinyon pines

**DOI:** 10.1111/j.1365-2699.2007.01786.x

**Published:** 2008-02

**Authors:** Kenneth L Cole, Jessica Fisher, Samantha T Arundel, John Cannella, Sandra Swift

**Affiliations:** 1 USGS Southwest Biological Science Center PO Box 5614, Flagstaff, AZ 86011, USA; 2 Environmental Sciences & Policy Program, Northern Arizona University PO Box 6077, Flagstaff, AZ 86011, USA; 3 Department of Geography, Planning, and Recreation, Northern Arizona University PO Box 15016, Flagstaff, AZ 86011, USA; 4 Flagstaff Area National Monuments, National Park Service 6400 N HWY 89, Flagstaff, AZ 86004, USA; 5 Quaternary Sciences Program, Northern Arizona University PO Box 4099, Flagstaff, AZ 86011, USA

**Keywords:** Climate modelling, needle anatomy, *Pinus edulis*, *Pinus monophylla*, pinyon pines, species climate window, western North America

## Abstract

**Aim:**

The geographical extent and climatic tolerances of one- and two-needled pinyon pines (*Pinus* subsect. *Cembroides*) are the focus of questions in taxonomy, palaeoclimatology and modelling of future distributions. The identification of these pines, traditionally classified by one- versus two-needled fascicles, is complicated by populations with both one- and two-needled fascicles on the same tree, and the description of two more recently described one-needled varieties: the *fallax*-type and *californiarum*-type. Because previous studies have suggested correlations between needle anatomy and climate, including anatomical plasticity reflecting annual precipitation, we approached this study at the level of the anatomy of individual pine needles rather than species.

**Location:**

Western North America.

**Methods:**

We synthesized available and new data from field and herbarium collections of needles to compile maps of their current distributions across western North America. Annual frequencies of needle types were compared with local precipitation histories for some stands. Historical North American climates were modelled on a *c*. 1-km grid using monthly temperature and precipitation values. A geospatial model (ClimLim), which analyses the effect of climate-modulated physiological and ecosystem processes, was used to rank the importance of seasonal climate variables in limiting the distributions of anatomical needle types.

**Results:**

The pinyon needles were classified into four distinct types based upon the number of needles per fascicle, needle thickness and the number of stomatal rows and resin canals. The individual needles fit well into four categories of needle types, whereas some trees exhibit a mixture of two needle types. Trees from central Arizona containing a mixture of *Pinus edulis* and *fallax*-type needles increased their percentage of *fallax*-type needles following dry years. All four needle types occupy broader geographical regions with distinctive precipitation regimes. *Pinus monophylla* and *californiarum-*type needles occur in regions with high winter precipitation. *Pinus edulis* and *fallax*-type needles are found in regions with high monsoon precipitation. Areas supporting *californiarum*-type and *fallax*-type needle distributions are additionally characterized by a more extreme May–June drought.

**Main conclusions:**

These pinyon needle types seem to reflect the amount and seasonality of precipitation. The single needle fascicle characterizing the *fallax* type may be an adaptation to early summer or periodic drought, while the single needle of *Pinus monophylla* may be an adaptation to summer–autumn drought. Although the needles fit into four distinct categories, the parent trees are sometimes less easily classified, especially near their ancestral Pleistocene ranges in the Mojave and northern Sonoran deserts. The abundance of trees with both one- and two-needled fascicles in the zones between *P. monophylla*, *P. edulis* and *fallax*-type populations suggest that needle fascicle number is an unreliable characteristic for species classification. Disregarding needle fascicle number, the *fallax*-type needles are nearly identical to *P. edulis*, supporting Little’s (1968) initial classification of these trees as *P. edulis* var. *fallax,* while the c*aliforniarum-*type needles have a distinctive morphology supporting Bailey’s (1987) classification of this tree as *Pinus californiarum*.

## Introduction

The pinyon pines (*Pinus* subsect. *Cembroides*) are small trees of semi-arid habitats of western North America. Two of the five to eight species currently recognized in the western United States have extremely widespread distributions: *Pinus monophylla* Torr. & Frem. (single-needle pinyon) and *Pinus edulis* Engelm. (Colorado pinyon). These two species dominate most of the western pinyon–juniper woodland and are of tremendous ecological importance. Although these two species are closely related and may hybridize, most of their populations occupy distinctly different climatic regimes. These one- and two-needled pinyon pines have been the focus of recent controversies regarding their taxonomic status, conservation attempts to both restrict and restore their range, their significance in palaeoclimatic reconstructions and projections of future distributions resulting from climate change (Cole *et al.*, 2007). In this paper we classify individual needles according to their anatomy, collate available information on the geographical distributions of these pines at the level of needle morphology rather than species, and model the climatic ranges occupied by these needle types. Our results have implications for the classification of these species and their varieties, and suggest a reinterpretation of their climatic tolerances.

## Contradictory classifications

The history of the classification of pinyon pines has largely been one of successive splitting. *Pinus monophylla* has been described as *P. edulis* var*. monophylla*, whereas *P. edulis* has been described as *P. monophylla* var. *edulis*. Both have been considered to be varieties of *Pinus cembroides* (Chronquist *et al.*, 1972). During most of the last century, *P. monophylla* was traditionally easily distinguishable from *P. edulis* by having a single needle in each fascicle rather than a pair. More recently, this easy dichotomy has been complicated by the description of two additional less well-accepted types with single-needle fascicles: the Arizona singleleaf pinyon (*P. edulis* var. *fallax*; Little, 1968), and the California singleleaf pinyon (*Pinus californiarum*; Bailey, 1987). Because the purpose of this paper is to study these pines from the perspective of the individual needles rather than any pre-conceived taxonomic scheme for the parent trees, we shall refer to the needles of these rarer types as the *fallax*-type and the *californiarum*-type.

The fascicle needle number character breaks down in many regions between populations, where individual trees can have a variable number of needles per fascicle; this has been variously described as hybridization, introgression or backcrossing between the one- and two-needled populations (Lanner, 1974; Lanner & Phillips, 1992; LaHood, 1995). Microhabitat has also been suggested as an influence on needle number (Welsh *et al.*, 1993).

Fascicle needle number also seems to be influenced by annual climatic fluctuations. The ratio of one- to two-needled fascicles has been shown to vary between *P. edulis* and *P. monophylla* type needles in at least one population in Nevada, reflecting precipitation over the previous 9 months (Tausch & West, 1986). A greater number of single-needle fascicles was found following a dry period. Similarly, trees in the transitional zones between *P. edulis* and the *fallax*-type populations in the Verde Valley of central Arizona also have variable ratios of one- to two-needle fascicles from year to year, which seem to correlate with precipitation over the prior year.

Little (1968) originally described the single-needled trees in central and north-western Arizona (Arizona singleleaf pinyon) as *P. edulis* var. *fallax*. Subsequently, Bailey (1987) subdivided all the single-needle pinyons into three different entities having variable colour, differences in the fascicle sheath and differences in needle morphology expressed as thickness, number of resin canals and stomatal rows. In his analysis, most remained as *P. monophylla*, but the south-western populations, occurring primarily in the coastal ranges of southern California and Baja California (California singleleaf pinyon), became *P. californiarum*, while the Arizona populations became *P. californiarum* subsp. *fallax.*

On the basis of monoterpene content, Zavarin *et al.* (1990) suggested that all the single-needle pinyons remain as *P. monophylla* but with the separation of three subspecies: *P. monophylla* subsp. *monophylla*, subsp. *fallax* (Little) Zavarin, and subsp. *californiarum* (D. K. Bailey) Zavarin. Lanner (1974) supported this classification of all single-needle trees as subspecies of *P. monophylla*, mostly on the premise that because this single-needled character was so rare in pines, only occurring in western North America, that it was unlikely to have evolved more than once. Lanner & Phillips (1992) described the populations with mixed needle types as ‘backcrosses’, where pollen from one type had cross-fertilized the other type.

These results were later contradicted by a study of pinyon chloroplast DNA (LaHood, 1995), which concluded that *P. monophylla* was distinct from all of the other varieties. *Pinus edulis* supported numerous similar plastid types including many that were shared with all *fallax*-type populations and one shared with a *californiarum*-type population. Because LaHood had distinguished taxa on the basis of needle fascicle number, he explained the close intermixing of plastids between *P. edulis* and the *fallax*-type as due to ‘introgression’ between the two different species. These plastid types shared between *P. edulis* and the *fallax*-type ranged throughout almost the entire range of both taxa, suggesting that most trees in Utah, Arizona and New Mexico are within the proposed ‘introgression zone’.

Despite their opposing conclusions, nearly every one of the studies listed above has emphasized that these pines are all very closely related. Regardless of their taxonomic classification, the purpose of this paper is to examine the geographical distribution of individual needle anatomical types and the correlations between needle type and climate. While needle anatomy may or may not be taxonomically meaningful, it is clearly related to climate. Trees in mediterranean (winter rainfall) climatic zones have stout needles with a thickened schlerophyll layer and more resin canals. In all accounts, single-needle fascicles are dominant on trees from the more arid habitats and times.

## Methods

### Measurement of needles

Measurements were taken on needles from dried herbarium specimens on loan from several herbaria and the authors’ collections. These specific needle characteristics and the use of dried needles (rather than fresh collections) were selected because they could be quantified with both modern herbarium collections and fossil needles from packrat middens. The mean and standard deviations were calculated for the following needle characteristics on six or more needles from each tree:

1. two counts of stomatal rows from the needle midsection and 5 mm to one side were averaged. A third count to the other side of the midsection was included in the average if the first two differed,
2. maximum and minimum needle widths using each needle cross-section were measured to the nearest micrometre at 200× on a compound microscope with a micrometer and averaged. On *P. edulis* specimens, the width of the needle across the crescent-shaped section was averaged with the thickness at the centre of the crescent,
3. count of the resin canals in microscopic cross-section.

Microscopic cross-sections were prepared on the needles using two different methods. Well-preserved, stout needles (usually *P. monophylla* and *californiarum*-type) could usually be sectioned with a razor blade after 24 h soaking in distilled H_2_O. Hand sectioning and viewing under a dissecting microscope at 60× often did not produce sufficiently clean sections or adequate resolution for reliable analyses, so these sections were illuminated from above using fibreoptic lights and viewed on a compound light microscope at 100×.

Better sections of the thin and brittle *P. edulis* and *fallax*-type needles could be obtained by pre-soaking specimens in a series of potassium hydroxide, ethyl alcohol and tertiary butyl alcohol, and embedding them in paraffin before microtome sectioning. These more delicate needles still tended to tear when sectioned, but the number of resin canals is usually recognizable even in incomplete sections by the presence of the characteristic thin-walled epithelial cells lining the resin canals (Gabilo-Hurd, 1980).

### Discrimination between the four needle types

**Figure 1:**
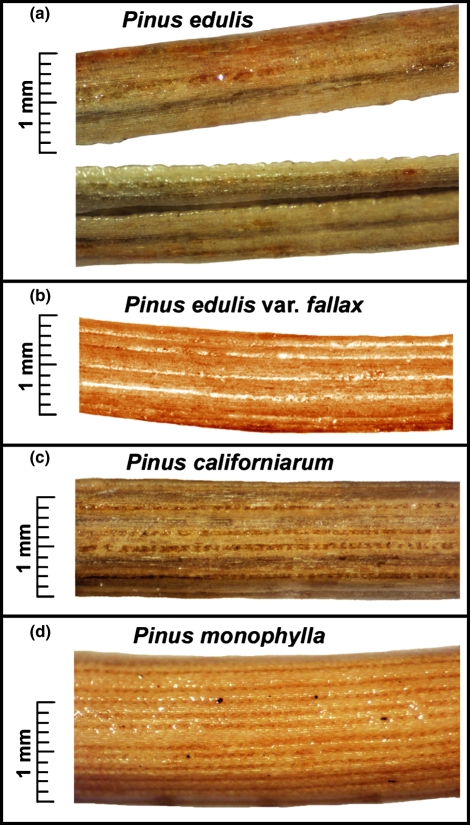
Surface images of needles typical of each type showing stomatal rows and diameters. (a) *Pinus edulis* needle fascicle from 2400-m elevation near Flagstaff, AZ. Image shows inside and outside of two needles in fascicle pair. (b) A *fallax*-type needle from 1100-m elevation near Camp Verde, AZ. (c) A *californiarum*-type needle from 1250-m elevation in the Santa Rosa Mountains, CA. (d) *Pinus monophylla* needle from 2000 m in the Eleana Range, NV.

The following working key based on measurements from this effort and previous results of Bailey (1987) was used for the delineation of these four anatomical needle types shown in Fig. 1.

#### Key to the needle types

Colorado pinyon (*P. edulis*) – fascicles contain two needles resulting in needles that are crescent-shaped in cross-section. Needles are thin, 0.8–1.1 mm average diameter when dried, contain two or three resin ducts and 8–15 stomatal lines.

Arizona singleleaf pinyon (*fallax*-type) – fascicles contain one needle, needles are thin, 0.8–1.2 mm in diameter when dried, contain two or three resin ducts and 8–16 stomatal lines.

Single-needle pinyon (*P. monophylla*) – fascicles contain one needle, needles are stout, 1.3–1.7 mm in diameter when dried, contain two to seven resin ducts and 17–30 stomatal lines.

California singleleaf pinyon (*californiarum*-type) – fascicles contain one needle, needles are stout, 1.2–1.6 mm in diameter when dried, contain 8–16 resin ducts and 13–18 stomatal lines.

### Needle-fascicle ratios in the Verde Valley

In order to examine any effects of climate on needle morphology in the proposed introgression zone between *P. edulis* and *fallax*-type (LaHood, 1995), we compared annual needle cohorts with recent climate at three locations in the Verde Valley of Arizona. Four growing shoots (north, south, east and west) were collected from each of five randomly selected trees from each of three pinyon stands, each within 200 m of a permanent climate station. Each shoot was cut into yearly growth increments for the 6- to 8-year period that the needles were retained. The percentage of one-needled vs. two-needled fascicles was computed for all fascicles (>10,000 needles in total) on each yearly shoot increment and compared with recorded precipitation at the adjacent climate station.

### Developing geographical range maps for these needle-types

**Figure 2:**
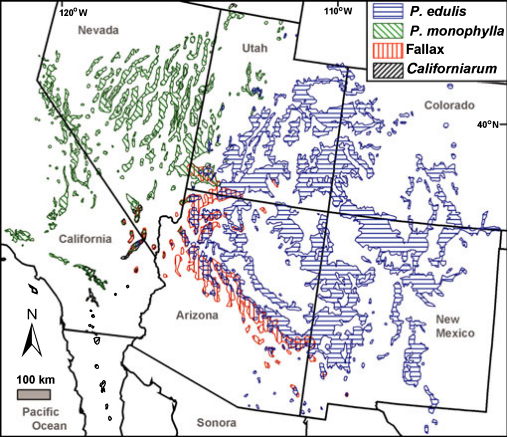
Current distribution ranges of one- and two-needled pinyon pine needle types based on data from Cole *et al.* (2003).

Maps depicting the geographical ranges of *P. edulis*, *P. monophylla*, *fallax*-type and *californiarum*-type needles were constructed by compiling available published maps and location data, herbarium specimens, observations of the authors, published elevational ranges and review comments from other field scientists (Fig. 2) (Cole *et al.*, 2003). The range of *P. edulis* was further refined using plot data (4307 presence records, 98,166 absence records) from the USDA forest inventory analysis program (Shaw *et al.*, 2005).

Most available detailed vegetation maps depict association types rather than species, and they can be misleading when applied to individual species (Cole & Cannella, 2005). For example, maps depicting the pinyon–juniper woodland were of little value for this study since the many juniper species cover a much wider range than pinyon. Also, mapping the *fallax* and *californiarum*-types was difficult since they have rarely been distinguished from the other types. Fortunately, both have limited ranges and maps could be generated using the geographical location data of Bailey (1987), new data developed during this study, field observations of the authors and review comments from other field scientists. Copies of these maps and data files, as well as additional details on the methods and data sources, are available at: http://sbsc.wr.usgs.gov/cprs/research/projects/global_change/RangeMaps.asp.

### Modern climate analysis

Modern monthly climate surfaces were created at *c*. 1-km resolution covering most of North America using the interpolation program anusplin (Hutchinson, 1989). The input data sets included monthly climate data from the Global Historical Climate Dataset (http://www.ncdc.noaa.gov/oa/climate/ghcn-monthly/index.php). The time period covered by this data set, encompassing most of the 20th century, was judged to be a more appropriate time span for comparison with slow-responding tree species distributions than other data sets using only 30-year mean values. The minimum monthly mean temperature (the one lowest monthly mean value recorded over the period of record), maximum monthly mean temperature (the one highest monthly mean value recorded over the period of record) and mean monthly precipitation were compiled for each climate station, producing one monthly value there for each of the three variables (36 values). Topographic data were added to climate stations from the GTOPO30 USGS data set and used as additional independent variables in calculating the interpolation algorithm. anusplin then used a thin-plate spline to calculate 36 monthly surfaces using the GTOPO30 data set values and matching its resolution. Various combinations of topographic data were tested for best performance. For this study, only elevation was included in the interpolation of the temperature data sets, while slope was also added to the precipitation interpolation. The resulting climate surfaces and calculation statistics are available at http://www.geog.nau.edu/global_change/climate_surfaces.html.

The modern climate ranges of the pinyon needle types were then determined by extrapolation of modern climates to each *c*. 1-km grid square within the modern range map for that type. Because minor errors in species ranges and georeferencing compound when multiple layers are overlain, the extreme tails of the species–climate distributions were eliminated by cutting off the highest and lowest 2.5% of the values.

Monthly temperature and precipitation extremes were grouped into five seasons (winter = December, January, February; spring = March, April; summer = May, June; monsoon = July, August, September; autumn = October, November). This seasonal distribution was designed to specifically represent climates of the arid south-western United States where spring often begins earlier than in colder regions, and the summer can be split between a very arid early summer and a very wet monsoonal late summer (Arundel, 2002, 2005; Cole & Arundel, 2007).

The relative importance of the 20 resulting seasonal climate variables (maximum and minimum mean monthly temperature and precipitation during the five seasons) was then evaluated using a new geospatial procedure, ClimLim (Arundel, 2002, 2005; Cole & Arundel, 2007) in order to quantify the effect of each variable in ‘limiting’ the distribution. First, the range of values within the spatial distribution for each pinyon type was calculated for all 20 variables. Next, applying a technique developed by Rod Hastings and first applied by Warren (1979), each pinyon type was ‘released’ one at a time from the confining influence of that variable. That is, the plant was allowed to disregard that one variable and expand its range until it was spatially confined by the remaining 19 variables. The relative significance of these variables was then ranked using the chi-square statistic comparing the amount of expansion resulting from the release of each variable. The assumption behind this ranking is that releasing a significant limiting variable will result in a much larger increase in range than releasing an incidental climate variable not controlling the plant’s spatial distribution.

Our species range maps depict areas of both species presence and absence, allowing the mapping of areas within the species’ climatic limits that are not currently occupied. This ‘potential climate range’ for each plant species (Jackson & Overpeck, 2000) acknowledges that ecological variables other than climate (i.e. fire, history, substrate, succession, migration) can be important in determining a species’ limits. Techniques employing only presence data cannot discriminate between these actual and potential ranges, instead mapping the entire potential range as occupied.

The values resulting from this analysis represent a ‘climate window’, within which ecological processes allow the species to persist. This climate window should not be confused with the usually much wider window under which a species may persist under artificial conditions, such as in a garden or growth chamber. These climate limits may be physiological, but more often they represent a less obvious climate-modulated variable such as fire frequency, interspecific competition, arthropod species or soil flora or fauna.

## Results

### Needle measurements

**Figure 3:**
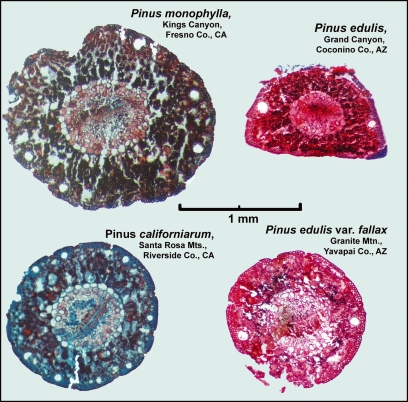
Representative cross-sections of four types of pine needles.

**Figure 4:**
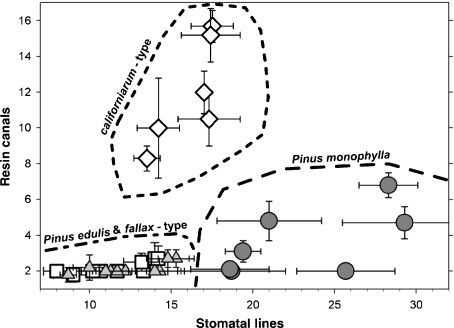
Plot of the mean and standard deviations of resin canal and stomatal row numbers from individual trees of the four needle types: filled circles, *P. monophylla*; filled triangles, *fallax*-type; hollow boxes, *P. edulis*; hollow diamonds, c*aliforniarum*-type.

**Figure 5:**
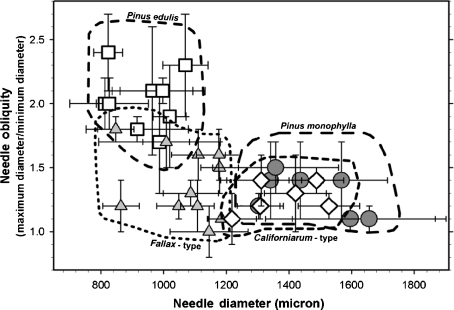
Plot of the mean and standard deviations of needle diameter and shape from individual trees of the four needle types: filled circles, *P. monophylla*; filled triangles, *fallax*-type; hollow boxes, *P. edulis*; hollow diamonds, *californiarum*-type.

The geographical extent of the four needle types calculated from the range maps and a *t*-test comparing the needle diameters are summarized in Table 1. Representative thin sections of needles are shown in Fig. 3 and the results of the pinyon needle measurements are shown in Figs 4 & 5. Because the preceding key to the needle types was progressively refined during the project, it correlates well with the results, as expected. However, the separation of needle types into four such distinct groups with few intermediate needles was unexpected. Even needles from trees containing a mixture of two needle types were classified distinctly into one group or the other, rather than intermediate between the two as would be expected. If hybridization of two species is occurring, it seems only to affect the tree as a whole. Individual needles on the trees usually fit as one of these four needle types regardless of their frequency on the tree.

**Table 1:** Geographical ranges and pine needle measurements. The significance of the difference from the other three types, as determined with a Student’s *t*-test, is shown.

Needle type	Total range (km^2^)	Mean needle diameter (μm)
*Pinus edulis*	205,091	941 ± 47**
*fallax*-type	37,354	1061 ± 141**
*californiarum*-type	5240	1357 ± 146*
*Pinus monophylla*	67,845	1484 ± 219*
*P. edulis/fallax*-type overlap	14,175	
*P. monophylla*/*P. edulis* overlap	1846	

* Exceeds 0.01 level

** exceeds 0.001 level.

Most trees from the regions where *P. edulis* intermingles with either *P. monophylla* or *fallax*-type yielded a mixture of both needle types on each tree, although sometimes only a small fraction of the needles differed from the predominant type. *Pinus edulis* and *fallax*-type needles were nearly identical in all features except for their round vs. crescent shape and consequent obliquity (roundness) resulting from the two-needled vs. one-needled fascicle (Figs 4 & 5). Both needle types were thin, had very few stomatal lines and usually contained two larger resin canals.

Needles of *P. monophylla* and *californiarum*-type were both stout with thickened sclerophyll layers and a series of smaller round resin canals arranged around their periphery, but they were surprisingly distinct in their numbers of resin canals versus stomatal lines (Fig. 4). No modern collections were observed where *P. monophylla* needles were intermixed with needles typical of *fallax*-type, or *californiarum*-type on the same tree. These combinations may exist, but if so they were not frequent enough to be observed during this study.

### Needle fascicle ratios in the Verde Valley

**Figure 6:**
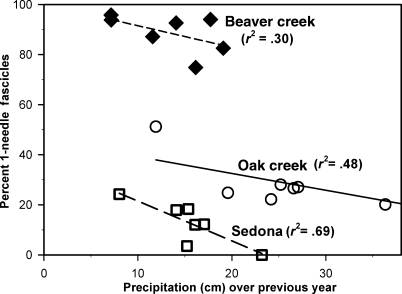
Percentage of one-needled fascicles on annual growth increments between 1998 and 2005 for three pinyon stands in the Verde Valley, AZ. Precipitation is totalled over the prior two wet seasons (from July of the previous year through to June of the current year). The stands are located adjacent to the Beaver Creek ranger station (filled diamonds; NCDC station no. 020670), Oak Creek Canyon (open circles; 026037) and the Sedona ranger station (open squares; 027708). Linear regressions and their associated *r*^2^ values are shown for each stand.

Needle fascicle ratios within three pinyon stands of the Verde Valley demonstrate a correlation between the number of single-needled (*fallax*-type) fascicles and low precipitation over the prior summer, autumn, winter and spring during which the needle cohort was forming and developing (Fig. 6). These results are very similar to those measured by Tausch & West (1986) on hybrids between *P. edulis* and *P. monophylla* in central Nevada, except that they are instead on mixtures between *P. edulis* and *fallax*-type needles. Also, like the results of Tausch & West (1986), certain individual trees within each stand appeared to be greatly influenced by climate, while other trees only changed slightly from year to year. These differences between trees may reflect genetic differences and/or microhabitat differences between the individual trees.

### Climatic ranges of needle types

**Figure 7:**
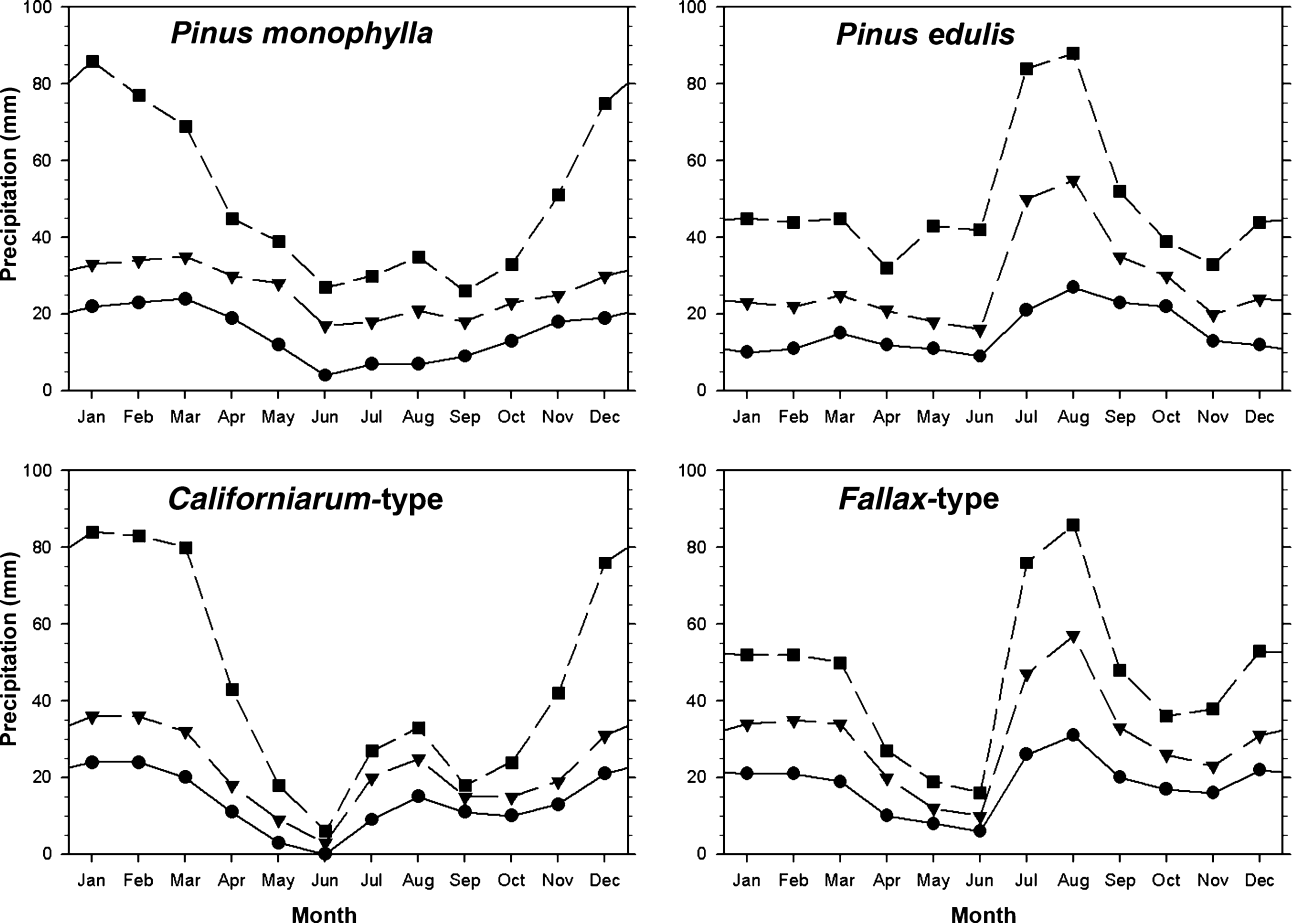
Summary of mean monthly precipitation values over the period of record of the Global Historical Climate Dataset modelled throughout the ranges of four needle types by km^2^. The total number of data points for each type by month is indicated by the range sizes listed in Table 1. Squares with short dashed line are the 95th percentile of all values; triangles with long dash the 50th percentile and circles with straight line the 5th percentile.

The range of monthly precipitation values for each needle type illustrates important differences in their ranges. *Pinus monophylla* and *californiarum*-type occupy areas with mostly winter precipitation (Fig. 7). *Pinus edulis* and *fallax*-type occupy areas with bi-seasonal precipitation with very high July–September (monsoon) precipitation. This conclusion was expected, but our analysis of monthly patterns also revealed that both *californiarum*-type and *fallax*-type occupy areas with distinctive early summer periods that are more arid than those of *P. monophylla* and *P. edulis*. Combined May and June precipitation over the ranges of these more restricted types averaged only 12 mm (*californiarum*-type) and 22 mm (*fallax*-type), while the larger ranges of the primary species averaged 45 mm (*P. monophylla*) and 34 mm (*P. edulis*) (Fig. 7). Because the ranges of these southerly types (*californiarum*-type and *fallax*-type) also have May–June temperatures ranging from 2–4°C warmer than the northern species (see Appendix S1 in Supplementary Material), evaporative stress on these southerly needles must be significantly higher during this dry period.

**Figure 8:**
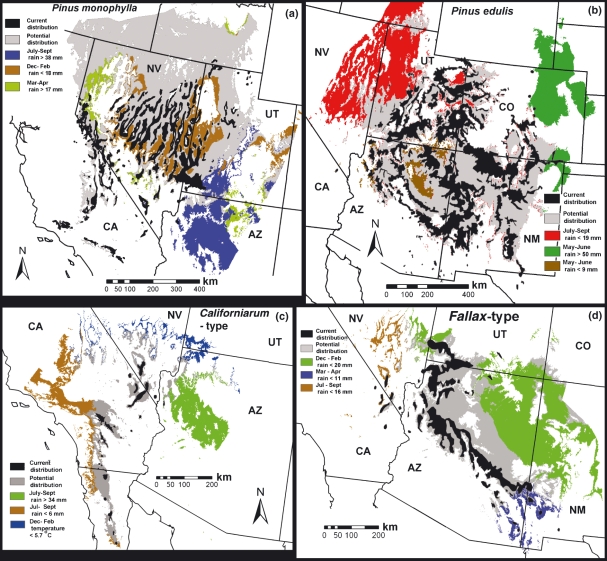
Maps showing the current distributions of pinyon needle types (black), potential climate regions (grey) and regions where they are excluded by their three most spatially significant limiting variables. (a) *Pinus monophylla*; limited by maximum monsoon (blue), minimum winter (brown) and minimum spring precipitation (green). (b) *Pinus edulis*; limited by minimum monsoon (red) and maximum (green) and minimum (brown) early summer precipitation. (c) The *californiarum-*type; limited by maximum (green) and minimum (brown) monsoon precipitation and minimum winter temperature (blue). (d) The *fallax*-type; limited by minimum winter precipitation (green) and maximum (blue) and minimum (brown) monsoon precipitation.

The geographical areas from which each species are limited by their three most significant variables are described below and illustrated in Fig. 8 (a)–(d). The complete climate windows and chi-square importance ranks for each needle type within each of the 20 seasonal extremes of temperature and precipitation are listed in Appendix S1 and contrasted with data applying to other climate data sets and other methods.

The ClimLim ranking of climate variables suggests that *P. edulis* is prevented from growing on the upper great plains of eastern Colorado, Wyoming and Nebraska (Fig. 8b) by amounts of early summer precipitation higher than 50 mm in either May or June. Although this limitation by high rainfall may seem counter-intuitive, it must be kept in mind that this limit applies to the entire matrix of climate-modulated species and processes within a natural ecosystem, rather than *P. edulis* in isolation. This limit may reflect the intolerance of *P. edulis* for the frequent fires sweeping through the grassland landscapes growing under this climate. *Pinus edulis* is prevented from expanding north-westward into the Great Basin areas occupied by *P. monophylla* by monsoon (July–September) precipitation below 19 mm. Conversely, *P. monophylla* (Fig. 8a) cannot expand south-eastward into the *P. edulis* areas of Arizona and southern Utah which have monsoon precipitation above 38 mm.

The area of potential climate for *P. monophylla* (Fig. 8a) extends far to the north of its actual distribution into Idaho and eastern Washington states. This may be the result of the high fire frequency on the grasslands of the Snake River Plains and its preference for the rocky outcrops of the Great Basin ranges. Also, the current distribution of *P. monophylla* may reflect the colder climates of the Little Ice Age, which ended about 150 years ago. It is possible that the tree’s distribution has not yet equilibrated to the warming climate since then. Although it is most obvious in this *P. monophylla* analysis, this pattern of potential late 20th century climates extending to the north and to higher elevations of observed distributions is evident in other species as well (Arundel, 2005; Cole & Arundel, 2007). *Pinus monophylla* is further limited by winter precipitation below 18 mm or spring precipitation below 17 mm in some inter-range valleys of the Great Basin.

The suitable climate for the *californiarum*-type (Fig. 8c) is in areas with monsoon rain between 6 and 34 mm. It is excluded from the wet monsoon areas of Arizona (the *fallax*-type range), but also the very dry monsoon parts of the California Coast Ranges (the *P. monophylla* range). The *californiarum*-type is the only pinyon population with a temperature variable ranked as one of the three most significant (Appendix S1). It is limited from the northern Mojave Desert (*P. monophylla* range) by winter minimum monthly mean temperatures below −5.7°C.

The area of suitable climate for *fallax*-type (Fig. 8d) is similar to that of *P. edulis* in that it is limited to the north-west by low monsoon precipitation (<16 mm). But it is further controlled by winter precipitation below 20 mm to the north-east in the Little Colorado River valley of Arizona and by spring precipitation below 11 mm in south-western New Mexico. This winter and spring precipitation may be required to offset the low May–June precipitation within its range (Fig. 7).

## Discussion

### Climatic patterns and needle anatomy

The results of these analyses suggest that the most crucial variable separating these needle types is the annual sequence of moisture availability. This should not be surprising since needles are leaves, and they should be expected to differentiate under variable moisture regimes. This result has implications for the study of plant–climate interactions, especially the reconstruction of past climates from fossil needles, or modelling future ranges of species that are classified by their leaves. Most efforts at climate modelling, whether modelling the past or the future, have made little effort to understand seasonality and instead have often modelled annual means, or perhaps January and July means. Modelling a July climate is particularly problematic for south-western North America, as it is typically a transition month between the early summer drought and the summer monsoon (Fig. 7).

Although the number of needles in a fascicle is easily determined, it appears to be a poor feature for species identification. In addition to the pinyon pines, other North American pines have flexible needle numbers in different bioclimatic regions. For example, needle number in *Pinus ponderosa* (ponderosa pine) can range from two, three or five, with infrequent fascicles ranging from one to eight. Like the pinyon, these pines have a tendency to reduce needle number on the same trees from three to two needles during dry periods (Haller, 1965).

Our climatic results for the *fallax*-type needle morphologies are of particular importance for palaeoclimatic reconstructions. These fossils had traditionally been identified as *P. monophylla*, based exclusively on needle fascicle number. They have been used to infer a mediterranean precipitation seasonality for the Sonoran Desert during the Pleistocene (Van Devender, 1990; Arundel, 2002) similar to that of the Great Basin today. The recent redesignation of many of these Sonoran Desert fossils as the *fallax*-type rather than *P. monophylla* (Lanner & Van Devender, 1998) should influence these palaeoclimatic reconstructions due to the drier, bi-seasonal precipitation regime over the modern *fallax* range (Fig. 7).

### Needle fascicle development

The number of resin canals and stomatal rows in each pine needle implies a possible model for climatic adaptation. Each single needle of *P. monophylla* has about the same number of resin canals, stomatal rows and needle biomass as the two-needle fascicle of *P. edulis*. This suggests that it could result from a *P. edulis* fascicle with the two individual needles merged (or vice versa). Furthermore, the *fallax*-type needle appears to be one-half of a *P. edulis* fascicle where one of the two needles failed to develop or was aborted. This scenario would seem to give *P. edulis* two alternative pathways for reducing evaporative stress by reducing needle surface area in environments with seasonal drought.

The first drought adaptive scenario – merging the two *P. edulis* needles into one *P. monophylla* needle – could assist in mediterranean climatic zones. This might be a similar but more complete process to that which merges the three-needle fascicles of the Mexican species, *Pinus nelsonii*, to form single-needle fascicles (Perry, 1991). *Pinus monophylla* and *californiarum*-type needles, with thick sclerophylous layers, waxy surfaces and abundant resin canals, are similar to the leaves of other shrubs in mediterranean zones and must be well adapted for this climate type. These smaller trees, especially the *californiarum*-type of southern California, often have the appearance of tall chaparral shrubs rather than trees.

The second scenario, dropping one of the *P. edulis* needles to form a *fallax*-type fascicle, may be a better adaptation to the early summer drought or periodic seasonal drought of central and northern Arizona. This mechanism may be flexible enough that some genotypes in the marginal zones between the pure *P. edulis* and *fallax*-type stands can respond by altering their frequency of needle types quickly after an especially wet or dry period (Fig. 6).

This model of the anatomical development of needles could explain the riddle presented by our results. While the needles fit into four distinct types, two different needle types can often be found on individual trees. Indeed, the classification of needles seems more straightforward than the classification of the parent trees.

Both of these scenarios are described as if these two single-needled pines independently developed from an ancestral *P. edulis*. Although *P. edulis* could be the ancestral pine, this is based on the unproven assumption that the ancestral species is still extant. There is no palaeobotanical support for any evolutionary scenario. All three needle types (*P. monophylla*, *P. edulis* and *fallax*-type) are found in deposits over 40,000 years old (K. L. Cole, unpublished data). Before this time, palaeobotanical records for these arid regions are particularly poor. Thus, the door is still open to many speculative evolutionary scenarios for these pines, just so long as they conclude prior to 40,000 years ago.

These scenarios described above are in direct conflict with the evolutionary paradigm of Lanner (Lanner, 1974; Lanner & Phillips, 1992), for which the controlling principle is that all single-needle pinyon pines must be descendants of the first single-needle pine, assumed to be *P. monophylla*. Our results seem to be best explained using the opposite approach to pine classification. Rather than applying needle fascicle number as the primary taxonomic feature, we have applied it as the least important feature. This perspective allows a more direct understanding of the vast intermixed zones between the *P. edulis* and *fallax*-type needles. If these trees are actually just leaf morphological variants of the same species as originally described by Little (1968), there is less need to invoke the mechanisms of backcrossing, introgression or hybridization. This perspective offers a much better explanation of their distributions, climatic affinities and chloroplast genetics (LaHood, 1995).

### Migration and differentiation

Every line of evidence suggests that these pines are all very closely related. The variability and plasticity in modern populations, and the contrasting taxonomic schemes of various authors, could be viewed as evidence that the traditional dichotomous taxonomy may be inappropriate for such a closely related, but diverse, species complex.

The core ancestral region for these pines appears to be a band stretching across the Mojave Desert across southern Nevada to the Mogollon Rim of central Arizona. Needles of three of the types (*P. monophylla*, *P. edulis* and the *fallax*-type) were present across the broad lowlands of this region throughout the late Pleistocene in fossil packrat middens. Many of these fossil assemblages contain mixtures of two needle types (K. L. Cole, unpublished data). Today this area lies just to the south of the intermixed zones where *P. edulis* overlaps with *P. monophylla*, and *P. edulis* overlaps with the *fallax*-type populations (Fig. 2). The higher mountains in the centre of this area, such as the Spring and New York Mountains, still support mixtures of all four needle types (Fig. 2). The populations of these desert ranges are so complex that Bailey (1987) identified them based upon the predominant needle type in each stand, rather than attempting to classify individual trees to species.

As climates warmed, starting about 11,600 years ago (Cole & Arundel, 2005), *P. edulis* populations expanded north-eastward, while *P. monophylla* populations expanded north-westward (K. L. Cole, unpublished data). The Holocene history of the rarer *fallax*-type and *californiarum*-type populations is poorly known. These larger populations of *P. edulis* and *P. monophylla* continued expanding throughout the Holocene, only reaching their north-western, northern, north-eastern and eastern limits within the last 1500 years (Betancourt *et al.*, 1991; Feiler, 1994; Nowak *et al.*, 1994; Gray *et al.*, 2006).

**Figure 9:**
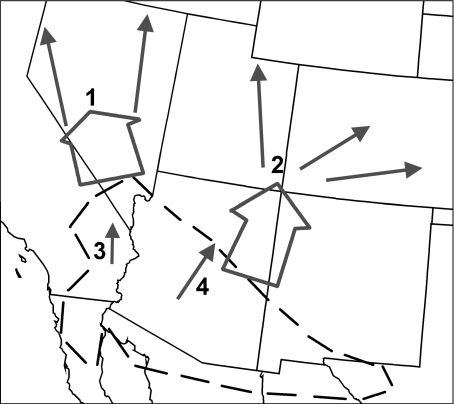
Conceptual diagram of the migration of pinyon types northward from their core region (dashed line) over the last 11,600 years: 1, *P. monophylla*; 2, *P. edulis*; 3, *californiarum* type; 4, *fallax* type.

As the populations of the *P. edulis* and *P. monophylla* genotypes migrated northward, they would have faced successive bottlenecks at their leading edges. This would have resulted in the pattern of broad distributions of two differentiated types in the north and more complex mixtures to the south, such as the *fallax* and *californiarum* types (Fig. 9). This is analogous to the post-glacial colonization of Europe by deciduous oaks (Dumolin-Lapégue *et al.*, 1997), the expansion of lodgepole pine into western Canada (Cwynar & MacDonald, 1987) and the expansion of five angiosperms and a fern from the Pacific Northwest of the USA into British Columbia (Soltis *et al.*, 1997). In all of these examples, a subset of the pre-existing genotypes expanded northward in the Holocene, leaving a more complex mixture of genotypes behind in the south.

## Conclusions

The four distinct needle types reported here reflect the seasonality of precipitation. The anatomy of the single-needle types is likely to represent drought adaptation through the reduction of surface area. In addition, these different populations can be shown through the spatial statistics of the ClimLim model to be geographically distributed according to precipitation regime.

The abundance of trees with both one- and two-needled fascicles in the zones between *P. monophylla, P. edulis* and *fallax*-type populations suggest that needle fascicle number is an unreliable characteristic for species classification. Disregarding needle fascicle number, the *fallax*-type needles are identical to those of *P. edulis*, supporting Little’s classification of these trees as *P. edulis* var. *fallax* (Little, 1968). The *californiarum-*type needles we studied have a distinctive needle morphology, supporting Bailey’s (1987) classification of this tree as *P. californiarum*.

The northward expansion of *P. monophylla* and *P. edulis* out of a diverse species complex over the last 11,600 years may have led to the fairly homogenous populations across the majority of their ranges in northern California, Utah and Colorado. Meanwhile, the more restricted populations remaining in southern California, Arizona, New Mexico and western Texas seem more diverse, supporting the *fallax* and *californiarum*-types, and possibly other rare genotypes as well.

## SUPPLEMENTARY MATERIAL

The following supplementary material is available for this article online:

Appendix S1 A comparison of different climate envelopes. Seasonal temperature and precipitation ranges occupied by pinyon types derived in this study are listed and compared with similar values derived using other climate data sets and studies.

This material is available as part of the online article from: http://www.blackwell-synergy.com/doi/abs/10.1111/j.1365-2699.2007.01786.x

## Biosketches

**Kenneth L. Cole** is a research ecologist with the US Geological Survey who has concentrated his research on vegetation change as caused by climate and other ecological processes. Much of his research has been on changing plant distributions of western North America as determined from the analysis of plant macrofossils contained within packrat middens.

**Jessica Fisher** completed a masters degree in the Environmental Sciences and Policy Program at Northern Arizona University and has worked extensively with plant taxonomy and anatomical studies of needles of pinyon pines.

**Samantha Arundel** is an associate professor in the Department of Geography, Planning and Recreation at Northern Arizona University and has worked extensively with climate modelling of plant species using geospatial systems.

Editor: Glen MacDonald

## References

[b1] Arundel ST (2002). Modelling climate limits of plants found in Sonoran Desert packrat middens. Quaternary Research.

[b2] Arundel ST (2005). Using spatial models to establish climatic limiters of plant species’ distributions. Ecological Modelling.

[b3] Bailey DK (1987). A study of *Pinus* subsection *Cembroides* I: The single-needle pinyons of the Californias and the Great Basin. Notes from the Royal Botanic Garden Edinburgh.

[b4] Betancourt JL, Schuster WS, Mitton JB, Anderson RS (1991). Fossil and genetic history of a pinyon pine (*Pinus edulis*) isolate. Ecology.

[b5] Chronquist A, Holmgren A, Holmgren N, Reveal J (1972). Intermountain flora: vascular plants of the Intermountain West, USA.

[b6] Cole KL, Arundel ST (2005). Carbon isotopes from fossil packrat pellets and elevational movements of Utah agave plants reveal Younger Dryas cold period in Grand Canyon, Arizona. Geology.

[b7] Cole KL, Arundel ST, Starratt SW, Cornelius P, Joelson JG (2007). Modeling the climatic requirements for Southwestern plant species. Proceedings of the Twenty-First Annual Pacific Climate Workshop.

[b8] Cole KL, Cannella J, van Riper C, Mattson D (2005). Species-based vegetation mapping: an example from the Grand Canyon. The Colorado Plateau II: Cultural, biological, and physical research.

[b9] Cole KL, Ferguson G, Cannella J, Spellenberg R, Saunders A, Arundel S, Riser J (2003). Digital rangemaps for North American one and two-needled pinyon pines (*Pinus monophylla, P. edulis,* and *fallax* and *californiarum*-types).

[b10] Cole KL, Ironside K, Arundel S, Duffy P, Shaw J, van Riper C, Sogge M (2007). Modeling future plant distributions on the Colorado Plateau: an example using *Pinus edulis*. The Colorado Plateau III: Integrating research and resources management for effective conservation.

[b11] Cwynar LC, MacDonald GM (1987). Geographical variation of lodgepole pine in relation to population history. The American Naturalist.

[b12] Dumolin-Lapégue S, Demesure B, Fineschi S, Le Corre V, Petit RJ (1997). Phylogeographic structure of white oaks throughout the European continent. Genetics.

[b13] Feiler EJ (1994). A 6,000 year record of vegetation change from West Carrizo Canyon, southeastern Colorado.

[b14] Gabilo-Hurd EM (1980). Ultrastructure and histochemistry of development in the needle of Pinus monophylla.

[b15] Gray ST, Betancourt JL, Jackson ST, Eddy RG (2006). Role of multidecadal climate variability in a range extension of pinyon pine. Ecology.

[b16] Haller JR (1965). The role of 2-needle fascicles in the adaptation and evolution of ponderosa pine. Brittonia.

[b17] Hutchinson MF (1989). A new objective method for spatial interpolation of meteorological variables from irregular networks applied to the estimation of monthly mean solar radiation, temperature, precipitation and windrun. CSIRO, Australia, Division of Water Resources Technical Memorandum.

[b18] Jackson S, Overpeck J, Erwin DH, Wing SL (2000). Responses of plant populations and communities to environmental changes of the Quaternary. Deep time, paleobiology’s perspective.

[b19] LaHood E (1995). A chloroplast DNA phylogeny of nine taxa in Pinus subsection Cembroides.

[b20] Lanner RM (1974). Natural hybridization between *Pinus edulis* and *Pinus monophylla* in the American Southwest. Silvae Genetica.

[b21] Lanner RM, Phillips AM (1992). Natural hybridization and introgression of pinyon pines in northwestern Arizona. International Journal of Plant Sciences.

[b22] Lanner RM, Van Devender TR, Richardson DM (1998). The recent history of pinyon pine in the American southwest. Ecology and biogeography of Pinus.

[b23] Little EL (1968). Two new pinyon varieties from Arizona. Phytologica.

[b24] Nowak CL, Nowak RS, Tausch RJ, Wigand PE (1994). Tree and shrub dynamics in northwestern Great Basin woodland and shrub steppe during the Late-Pleistocene and Holocene. American Journal of Botany.

[b25] Perry JP (1991). The pines of Mexico and Central America.

[b26] Shaw JD, Steed BE, DeBlander DT (2005). Forest Inventory Analysis (FIA) annual inventory answers to the question: what is happening to pinyon-juniper woodlands?. Journal of Forestry.

[b27] Soltis DE, Gitzedanner MA, Strenge DD, Soltis PS (1997). Chloroplast DNA intraspecific phylogeography of plants from the Pacific Northwest of North America. Plant Systematics & Evolution.

[b28] Tausch RJ, West NE, Everett RL (1986). Morphological variation/precipitation relationships of Great Basin single-needled pinyon. Proceedings of the Pinyon-Juniper Conference, January 13–16, 1986, Reno, NV.

[b29] Van Devender TR, Betancourt JL, Van Devender TR, Martin PS (1990). Late Quaternary vegetation and climate of the Sonoran desert, United States and Mexico. Packrat middens: the last 40,000 years of biotic change.

[b30] Warren DK (1979). Precipitation and temperature as climatic determinants of the distribution of Fouquieria columnaris.

[b31] Welsh SL, Atwood ND, Goodrich S, Higgins LC (1993). A Utah flora.

[b32] Zavarin E, Snajberk K, Cool L (1990). Chemical differentiation in relation to the morphology of the single-needle pinyons. Biochemical Systematics and Ecology.

